# Parental Vaccine Hesitancy, Trust in Physicians, and Future Vaccination Intentions: A PACV Cross-Sectional Study

**DOI:** 10.3390/vaccines13111127

**Published:** 2025-11-01

**Authors:** Sandra Janiak, Elwira Piszczek, Agnieszka Buczkowska, Krzysztof Buczkowski

**Affiliations:** 1 Department of Family Medicine, Nicolaus Copernicus University in Torun, Collegium Medicum in Bydgoszcz, 85-094 Bydgoszcz, Poland; sandra.janiak@cm.umk.pl; 2 Institute of Sociology, Nicolaus Copernicus University in Torun, 87-100 Torun, Poland; elwirapi@umk.pl; 3 University Hospital in Poznan, Przybyszewskiego 49, 60-355 Poznan, Poland; buczkowskaa33@gmail.com

**Keywords:** parental attitudes, vaccine acceptance, healthcare communication, physician–patient relationship, preventive health behavior, immunization decision-making

## Abstract

Background/Objectives: Parents’ vaccine hesitancy constitutes a global challenge, strongly associated with trust in healthcare professionals. This study aimed to identify socio-demographic predictors of parental pro- and anti-vaccination behaviors and investigate the association between these factors and intentions regarding children’s future immunizations. Methods: We conducted a cross-sectional online survey using the Parent Attitudes about Childhood Vaccines (PACV) questionnaire. Participants were recruited purposively and via snowballing through parenting groups and educational institutions. Results: We surveyed 1.046 parents and provided attitudes for 1.701 children; 85.1% of child-specific responses reflected positive attitudes (PACV ≤ 50; median 16.7). In univariate regression, employment (OR = 2.172, 95% CI: 1.530–3.084; *p* < 0.001) and healthcare employment (OR = 2.907, 95% CI: 1.983–4.262; *p* < 0.001) increased the odds of positive attitudes, whereas each additional household member (OR = 0.693, 95% CI: 0.597–0.805; *p* < 0.001) and child (OR = 0.677, 95% CI: 0.579–0.792; *p* < 0.001) reduced them. Multivariable models confirmed higher odds for suburban/rural residence (OR = 1.614, 95% CI: 1.037–2.513; *p* = 0.034), employment (OR = 1.869, 95% CI: 1.284–2.721; *p* = 0.001), and healthcare employment (OR = 2.785, 95% CI: 1.872–4.144; *p* < 0.001). Among prior non-vaccinators (*n* = 114), 39.5% planned to vaccinate, 41.2% did not. Those who planned showed greater trust than non-planners in the child’s doctor (mean: 7.49 vs. 3.74) and schedule (mean: 7.89 vs. 1.40), *p* < 0.001, with lower trust correlating with greater hesitancy and safety concerns. Conclusions: Trust in physicians was strongly associated with both current attitudes and future intentions. Trust-focused, patient-centered communication may be related to more positive vaccination attitudes among parents. However, longitudinal research is needed to determine whether such trust could influence changes in vaccination behavior, especially since parents’ attitudes toward vaccinations can evolve with the arrival of subsequent children.

## 1. Introduction

Vaccination forms the foundation of global health security. Preventing infections reduces mortality, improves quality of life, and decreases the burden on healthcare systems [1]. It is one of the most important medical interventions that can positively affect the health of every individual. Since 1974, infant mortality has significantly declined worldwide, with vaccinations estimated to be responsible for 40% of this achievement [2]. Researchers estimate that between 1974 and 2024, vaccination programs targeting 14 modeled pathogens prevented 154 million deaths, including 146 million among children under five, of which 101 million were infants under one year of age [2]. These programs have significantly reduced the incidence of many diseases and, in some cases, led to their complete eradication, exemplified by smallpox. The last documented case of this disease occurred in Somalia in 1977, and three years later, the World Health Organization (WHO) declared the official eradication of smallpox [2]. For practicing physicians, carrying out vaccination-related tasks has become a significant challenge, especially as many people forgo vaccination in the context of improving epidemiological conditions. The success of vaccination programs has paradoxically led to complacency. Without direct experience of dangerous diseases such as smallpox, polio, or measles, it is more challenging to recognize their threats. The spread of misinformation about the harmfulness of vaccines, which contradicts scientific knowledge, further reinforces this process [3,4].

In addition, the Coronavirus Disease 2019 (COVID-19) pandemic intensified public discourse on this issue (radio, television, internet) and brought the discussion into family and social circles [5]. After this period, social attitudes toward vaccination have become more ambivalent [6].

In this context, parental trust in physicians as the primary source of vaccination information plays a crucial role in decision-making [7,8]. The global scale of vaccine hesitancy regarding childhood immunizations underscores the importance of this trust. Generally, vaccine hesitancy—defined by the WHO as a delay in accepting or refusing vaccines despite the availability of vaccination services—has been reported in more than 90% of countries worldwide [9].

Recent theoretical frameworks of vaccine behavior, such as the 5C model (confidence, complacency, constraints, calculation, and collective responsibility), help explain the psychological antecedents of vaccination decisions [10]. Within this framework, trust constitutes a key element of the confidence component, referring to the belief in the effectiveness and safety of vaccines and the competence and reliability of the healthcare system and its representatives [7,8].

Patient-centered communication is key, encompassing open-ended questioning, an empathetic approach, and active listening. These strategies foster long-term trust between healthcare providers and patients, as well as their caregivers. Research shows that high-quality communication not only fosters positive relationships but also translates into greater patient engagement in medical procedures and preventive actions, higher satisfaction with care, and better adherence to therapeutic recommendations, ultimately increasing the effectiveness of the treatment and prevention process [4,11].

In European healthcare systems, the qualification of children for vaccination is assigned either to a physician or a nurse, depending on the model of primary healthcare organization. This stage constitutes a crucial moment of contact with parents, as it is during the qualification process that their questions and concerns about vaccination are most frequently raised [12]. According to current procedures in Poland, every child scheduled for vaccination must undergo a qualifying medical examination [13]. The purpose of this qualification, which includes a targeted medical interview and physical examination, is to confirm the indications and exclude any contraindications for administering the vaccine. This stage also allows physicians to talk with parents, especially regarding doubts and concerns about vaccination. Limited time for this step, or skipping it altogether, may contribute to developing negative attitudes toward vaccination [8]. Previous studies have shown that parents’ trust in healthcare professionals, particularly physicians responsible for implementing vaccination programs, strongly predicts vaccine acceptance [7]. However, this trust is not constant; it develops under the influence of prior experiences. In a study published in 2008, the most significant proportion of parents who changed their minds about delaying or refusing a vaccination for their child identified “information or assurances from a healthcare provider” as the main reason [14]. Nevertheless, researchers still know little about how trust in physicians shifts earlier parental decisions against vaccination toward pro-vaccination choices, or whether parents who have previously refused vaccinations plan to maintain such behavior. Previous studies have rarely examined the potential for attitude change among parents who had previously refused vaccination. Understanding this conversion potential and the role of trust in distinguishing parents who reconsider vaccination decisions may offer important insights for designing targeted communication strategies that address hesitancy and strengthen trust within healthcare interactions. If not all do, the question remains as to what respects and whether they differ. To fill this gap, the present study systematically examined socio-demographic predictors of parents’ pro- and anti-vaccination behaviors and the associations between these factors and their intentions regarding future childhood vaccinations.

## 2. Materials and Methods

### 2.1. Study Design

This study was cross-sectional and conducted between 10 February 2024 and 19 November 2024 using an anonymous online questionnaire based on the Parent Attitudes about Childhood Vaccines (PACV) survey.

The complete questionnaire comprised 28 questions: 12 demographic questions (Q1–Q12), the central part of the PACV survey (Q13–Q27), and one open-ended question (Q28) that allowed respondents to share their own comments or observations on the topic under discussion (Table A1).

The PACV section consists of 15 disjunctive closed-ended questions presented in three formats: three trichotomous questions (yes/no/do not know), ten questions using a five-point Likert scale, and two questions using an eleven-point numerical scale. The questionnaire was distributed among target groups using various communication channels. Researchers recruited participants using purposive and snowball sampling. The researchers shared the form on parenting forums and groups on Facebook and emailed it to educational and childcare institutions such as schools, kindergartens, and nurseries. In addition, the researchers directly contacted educational institutions to enable distribution through electronic communication channels with parents (e.g., attendance registers, newsletters, internal platforms, parent groups).

### 2.2. PACV Questionnaire

The PACV survey is a validated tool successfully used in several countries to identify vaccine-hesitant parents [15,16,17,18,19,20,21,22]. It was developed in 2010 to identify parents with reduced acceptance of vaccinations, which may lead to delays or refusal to follow the recommended immunization schedule [23,24]. The original PACV questionnaire has been translated and validated in many countries, including Poland [25].

### 2.3. Statistical Analysis

Data entry and statistical analyses were performed using IBM SPSS Statistics (version 10.0 for Windows) and R (version 4.4.3). All analyses used a significance level of 0.05. Depending on the type of analysis, calculations relied either on the number of respondents (n = 1046) or on the number of children referenced in their responses (n = 1701). Missing responses accounted for discrepancies in reported values.

The PACV scoring protocol guided the interpretation of total scores. Each response carried a numerical value, and the sum of these values produced the raw score. The scoring system then converted the raw score into a percentile scale ranging from 1 to 100, where 0 indicated complete acceptance of vaccination and 100 indicated complete rejection as a preventive measure against infectious diseases. A final score above 50 points marked a high probability of vaccine hesitancy.

Univariate and multivariate logistic regression analyses (see Section 3.2.1) examined associations with potential predictors using data prepared using the PACV dichotomous scoring procedure. Results appear as odds ratios (OR) with 95% confidence intervals. The multivariate logistic regression models included variables that showed statistical significance or a trend toward significance (*p* < 0.20) in univariate analyses. Model fit was fair (Nagelkerke R^2^ = 0.09; Hosmer–Lemeshow χ^2^ = 6.53, *p* = 0.588).

**Figure 1:**
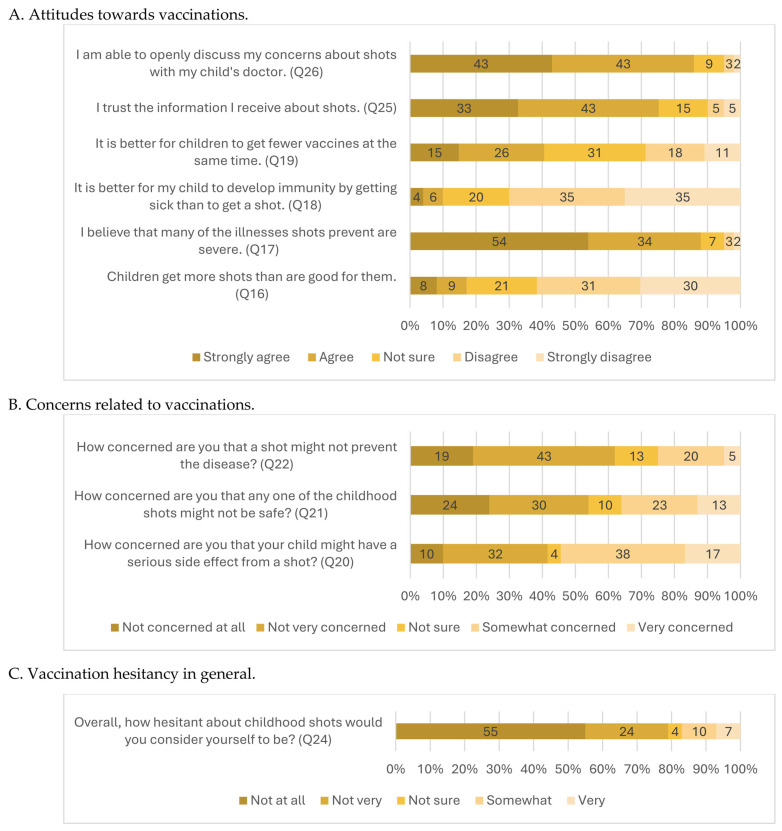
Percentage of responses regarding attitudes, concerns, and vaccination hesitancy in general. (**A**) Attitudes toward vaccinations; (**B**) Concerns related to vaccinations; (**C**) Vaccination hesitancy in general.

Univariate and bivariate analyses were also prepared (see Section 3.2.2 and Section 3.2.3). For a more straightforward presentation of data, the distribution of responses for selected variables was visualized in the form of graphs, taking into account differences in the wording of the responses (Q16, Q17, Q18, Q19, Q25, and Q26/Figure 1A; Q20, Q21, and Q22/Figure 1B; Q24/Figure 1C).

The analysis provided interpretations for the entire study sample and selected respondent subgroups. Statistical measures and significance tests matched the measurement levels of variables, group sizes, and variables’ distribution types. The Kruskal–Wallis H test, Spearman’s rho, and the Mann–Whitney U test supported the analyses.

We received limited open-ended responses (Q28) and did not conduct formal qualitative coding. We reviewed the comments descriptively to identify general themes and illustrative remarks.

## 3. Results

### 3.1. Participants

A total of 1046 respondents completed the survey. Mothers of children predominated (91.24%), as did individuals with higher education (86.04%) and residents of large cities (43.4%). In total, 40.44% of respondents lived in rural areas or small- and medium-sized towns. The mean age of participants was 36 years (range: 20–60 years). Households of three (40.63%) and four members (40.73%) accounted for the largest share, as did households with one (49.19%) or two children under 18 years of age (40.92%). The mean age of children in the surveyed families was 6.5 years, with the youngest being under one year of age. Most respondents were employed (89.29%), and 27.82% reported working in the healthcare sector. Detailed sociodemographic characteristics of the study group are presented in Table 1.

### 3.2. PACV—General Characteristics of the Index

Parents of more than one child could provide separate responses for each child; therefore, the overall PACV score was calculated for 1701 responses. The mean score on the 0–100 scale was 25.1 (Q1 = 6.67, Q3 = 36.67). Positive attitudes (≤50 points) predominated in the study group and were reported in relation to individual children by 85.07% of parents/caregivers (Table 2).

#### 3.2.1. Sociodemographic Characteristics and Attitudes Toward Vaccination

Univariate logistic regression models (separate for each variable considered) revealed significant associations (Table 3). Employment status more than doubled the likelihood of positive attitudes toward vaccination (OR = 2.172). Similarly, working in healthcare was associated with nearly a threefold increase in the probability of positive vaccination attitudes (OR = 2.907). In contrast, each additional household member reduced this likelihood by 30.7% (OR = 0.693), and each additional child by 32.3% (OR = 0.677).

#### 3.2.2. Vaccine Safety, Trust, and Vaccination-Related Behaviors in Respondents’ Opinions

Among the respondents, the vast majority (88%) had never decided against vaccinating a child for reasons other than illness or allergy. In contrast, 10.9% reported having made such a decision, with only three individuals making different choices for different children. Overall, 119 parents (11.4%) declared having delayed vaccination for reasons other than illness or allergy, and 46 (4.4%) reported doing so, but not for all of their children. At the same time, 921 respondents (88%) confirmed their willingness to vaccinate another child in the future, while 64 (6.1%) were uncertain about how they would proceed (Table 4).

The data show that respondents expressed the strongest conviction regarding the role of vaccines in preventing serious diseases: 54% were firmly convinced, and 34% were convinced. Respondents also strongly agreed with the possibility of openly discussing vaccination with their child’s doctor (43% strongly agreed and 43% agreed). General trust in vaccination information was somewhat lower, with 33% strongly agreeing and 43% agreeing. The most tremendous uncertainty concerned the number of vaccines administered during a single visit: 31% were unsure whether receiving fewer vaccines at one time was preferable. In comparison, 41% believed it was more beneficial. Only a small proportion of respondents agreed that children receive more vaccines than are good for them (8% strongly agreed, 9% agreed). Even fewer agreed that a child should acquire immunity through illness rather than vaccination (4% strongly agreed, 6% agreed) (Figure 1A).

Concerns about adverse events following immunization generated the highest levels of anxiety, with 55% reporting being somewhat or very concerned. In total, 36% of respondents expressed concern about vaccine safety. We observed the lowest level of concern regarding the perceived ineffectiveness of vaccination. A total of 25% of respondents expressed fears that vaccination might not prevent disease (Figure 1B).

Of all respondents, 55% reported no hesitancy toward vaccination, while 24% reported being somewhat hesitant. Only 7% indicated that they were very hesitant (Figure 1C).

Overall, the analyses indicated high trust in the child’s doctor and the recommended vaccination schedule. When allowed to respond on a 0–10 scale (with higher scores indicating greater trust), respondents confirmed that following the recommended vaccination schedule was suitable for their child (mean = 8.28) and that, overall, they trusted their child’s doctor (mean = 7.8) (Table 5).

#### 3.2.3. Relationships Between Trust, Hesitancy, and Current and Future Vaccination Decisions

Using the PACV questionnaire, we examined how trust in vaccinations and in the child’s doctor varied depending on parents’ declarations regarding past and planned vaccinations of their children.

The tests showed that low trust in the child’s doctor co-occurred with variables indicating low trust in vaccinations:

1. Among respondents who had previously decided not to vaccinate a child (for reasons other than illness or allergy), trust in the child’s doctor was the lowest. On the 0–10 scale (higher scores indicate greater trust), the mean score for non-vaccinating parents was 5.34, compared with 8.13 for vaccinating parents (Kruskal–Wallis H test, *p* < 0.001; data for Q27 and Q14).
2. Similarly, respondents who did not plan to vaccinate future children showed the lowest trust in the child’s doctor. On the 0–10 scale, the mean score was 3.95 for parents not planning vaccination, 8.24 for those planning vaccination, and 5.27 for those responding “I do not know” (Kruskal–Wallis H test, *p* < 0.001; data for Q27 and Q23).
3. Respondents with lower trust in the child’s doctor hesitated more about vaccination in general (Spearman’s rho = −0.416, *p* < 0.001; data for Q27 and Q24).
4. Respondents with lower trust in the child’s doctor also placed less trust in vaccination information (Spearman’s rho = −0.587, *p* < 0.001; data for Q27 and Q25).

The questionnaire also allowed us to examine how trust in the child’s doctor correlated with concerns about vaccine safety. Overall, lower trust in the child’s doctor was associated with greater concern about vaccination safety:

1. Respondents who were more concerned about vaccine safety reported being unable to openly discuss their concerns with the child’s doctor (Spearman’s rho = 0.294, *p* < 0.001; data for Q26 and Q21).
2. Respondents with lower trust in the child’s doctor reported higher concern about vaccine safety (Spearman’s rho = −0.461, *p* < 0.001; data for Q27 and Q21).
3. Respondents with lower trust in the child’s doctor expressed greater concern about adverse vaccine reactions (Spearman’s rho = −0.426, *p* < 0.001; data for Q27 and Q20).
4. Respondents who were more concerned about adverse reactions were also those unable to openly discuss their concerns with the child’s doctor (Spearman’s rho = 0.26, *p* < 0.001; data for Q26 and Q20).

Parents who had previously decided not to vaccinate a child (for reasons other than illness or allergy) reported higher levels of distrust and more doubts about vaccine safety and the vaccination schedule than parents who had never made such a decision. These parents were more likely to agree with the statements that

1. Children receive more vaccinations than are good for them (Q16).
2. Developing immunity through illness is better than through vaccination (Q18).
3. Receiving fewer vaccinations at a single visit is better (Q19). (For this analysis, we recoded Q14 into a dichotomous scale with the responses: “yes” and “no.” We eliminated eight “I do not know” responses and three responses of “it depends on the child.” We confirmed all conclusions using the Mann–Whitney U test, *p* < 0.001.)

They were also more likely to disagree with the statement that the diseases prevented by vaccination are serious (Q17), to distrust vaccination information (Q25), and to express greater hesitancy about childhood vaccinations (Q24) (For this analysis, we recoded Q14 into a dichotomous scale with the responses: “yes” and “no.” We eliminated eight “I do not know” responses and three responses of “it depends on the child.” We confirmed all conclusions using the Mann–Whitney U test, *p* < 0.001).

It is noteworthy, however, that parents who had previously decided not to vaccinate a child (for reasons other than illness or allergy) did not always intend to repeat this pattern. Almost 40% declared they would like a subsequent child to receive the recommended vaccination, while 19.3% were unsure. Only 41.2% declared they would maintain a non-vaccination approach for another child (Table 6).

Apparent differences in trust—particularly toward the child’s doctor and the vaccination schedule—emerged between parents/caregivers who had previously refused vaccination but (A) planned to vaccinate and those who (B) did not. Parents repeating the pattern of non-vaccination expressed far greater distrust. On a 0–10 scale (with higher values indicating greater trust), parents who had previously refused vaccination but planned to vaccinate reported a mean trust score of 7.49 for the child’s doctor, compared with 3.74 among those repeating the non-vaccination pattern. A similar trend appeared for trust in the vaccination schedule, where the differences were even more pronounced, with mean scores of 7.89 versus 1.40, respectively (differences statistically significant) (Table 6).

## 4. Discussion

In this study, which applied the PACV questionnaire, we aimed first to identify sociodemographic predictors of pro- and anti-vaccination behaviors and second to capture correlations between declared attitudes toward vaccination and views on its effectiveness and safety, with particular attention to issues of trust, especially in relation to healthcare providers.

In our sample, individuals with higher education clearly dominated (86.94%). However, education did not show a statistically significant effect on vaccination attitudes (*p* > 0.05). Referring to the Polish context, it is important to note that higher education prevails nationally in the age group we examined. Statistics Poland data show that in 2021, around 60% of Poles aged 25–44 had higher education. Moreover, in Poland, women more often hold higher education than men and constitute the vast majority of our study group [26]. The specificity of the sample may explain the lack of significant impact of education. Nevertheless, although many studies demonstrate a positive association between education and pro-vaccination attitudes [27,28,29,30,31], education operationalized as a diploma (as measured in PACV) increasingly appears insufficient for explaining vaccination attitudes. Studies show that a broader understanding of education—as an individual resource of competencies, but also as a dimension of national policies (e.g., mean and expected years of schooling, student–teacher ratios)—sometimes even reveals negative correlations, with higher education associated with more negative attitudes toward vaccination [32,33].

Our sample also displayed an overrepresentation of women compared to men, with mothers making up 91.24% of respondents. What seems crucial here is not reflecting the population structure but collecting opinions from those who manage health matters within families. Numerous studies show that women, particularly mothers, typically take responsibility for “medical issues” in the household, such as doctor visits and caring for sick family members. This division of roles is characteristic of Poland and many other countries [34,35,36,37,38,39,40,41,42,43,44,45,46]. We acknowledge that our sample does not represent the Polish population; nevertheless, the visible overrepresentation of women and individuals with higher education required additional commentary. Readers should consider this limitation when interpreting the results, because the purposive, digitally based recruitment may limit the generalizability of the findings to the broader population of Polish parents.

Other sociodemographic factors were also associated with pro-vaccination attitudes. Being employed, working in healthcare [27], and living in rural areas or in suburban zones of large cities were more likely to be associated with pro-vaccination attitudes than living in large cities [28]. By contrast, households with more children [31] and members showed a lower likelihood of pro-vaccination attitudes.

A clear predominance of pro-vaccination attitudes also characterized the study group. Using the standard PACV methodology, which defines scores of 0–50 as positive and 51–100 as negative, we observed that 85.07% of respondents fell into the positive category. Other descriptive statistics supported this finding: the median was 16.67 points, and the third quartile indicated that 75% of respondents scored below 37 points. We emphasize that our sample was not representative and that this is a characteristic of our specific, purposive sample. Nevertheless, referring to Polish statistics on vaccination rates for children under 18 years of age (from the current vaccination schedule), our sample does not differ from the national data. Despite a downward trend in vaccination rates, coverage among Polish children remains high. For example, in 2022, the first measles, mumps, and rubella (MMR) vaccination at age three reached 90.9%, while the booster at age 11 reached 86.1% [37]—despite MMR being one of the most debated vaccinations among parents.

Comparisons of sociodemographic factors with vaccine hesitancy across studies are difficult. Meta-analyses highlight differences in applied questionnaires and diverse socio-cultural, historical, and epidemiological contexts [38,39]. Another complicating factor is that most quantitative studies on parental vaccine hesitancy, like ours, are based on purposive samples. Although these studies provide valuable insights, the lack of representativeness hinders tracking long-term trends and generalizing findings to broader populations (e.g., at regional or national levels). Moreover, sociodemographic variables do not serve an explanatory function but merely help capture the specificity of a given sample [40,41]. Considering these issues, we opted for a non-standard use of the PACV questionnaire in statistical analyses—not limited to calculating the total score but also including exploratory analyses of relationships between individual PACV items and other variables—drawing on Data Mining approaches. This method allowed us to explore data creatively and detect unexpected associations [42]. In practice, we correlated PACV variables in bivariate tests and compared specific subgroups of respondents, focusing primarily on differences between those with distinct prior experiences and declared future vaccination intentions. Preliminary analyses highlighted physician-related variables (e.g., trust and the ability to talk openly with the child’s doctor) as particularly relevant, so we further correlated these with opinions on vaccine effectiveness, trust in vaccine information, safety concerns, adverse event following immunization (AEFI), and attitudes toward the vaccination schedule.

As mentioned, most parents in our sample expressed positive attitudes toward vaccination. Concerns were most pronounced around vaccine side effects, with over half (55%) expressing worry. Parents who reported lower trust in their child’s doctor and those unable to discuss concerns openly showed significantly higher levels of anxiety. These parents were also more likely to distrust vaccine information, worry about vaccine safety, and delay or refuse vaccination (for reasons other than illness or allergy), or to declare unwillingness to vaccinate future children. Other studies reported similar findings: parents with lower trust in obstetricians/gynecologists and children’s doctors often sought advice from alternative sources such as doulas, midwives, or lactation consultants [43]. This lack of trust also drives reliance on additional, often informal, information channels, bypassing physicians [44]. Research indicates that physician attitudes are strongly associated with parental trust and views on vaccination [8,45].

Our analyses also revealed that even parents who had previously refused vaccination remain open to dialogue and cooperation toward vaccinating in the future. Among the 114 respondents who declared they had not vaccinated their children for reasons other than illness or allergy, only 41.2% planned to continue this approach. A comparable proportion, 39.5%, intended to vaccinate their children in the future, while 19.3% remained undecided. Contrary to our expectations, most parents did not indicate a continuation of non-vaccination patterns. Importantly, those who did not rule out future vaccinations showed significantly higher trust in their child’s doctor and the vaccination schedule. Similar findings emerged in a Canadian study, demonstrating that maternal attitudes toward vaccination can evolve. Prior decisions not to vaccinate do not necessarily determine future choices. Instead, parental attitudes shifted under the influence of personal experiences, new information, and growing parenting confidence, often supported by positive interactions with healthcare providers, who played a vital role in this process [46].

This transparent trust gradient between parents who reconsider vaccination and those who persist in refusal highlights the practical value of trust-based, tailored communication strategies to support hesitant parents. Positioning these findings within the trust–vaccination nexus highlights that confidence, rather than mere information availability, plays a fundamental role in shaping vaccination intentions. This perspective extends beyond descriptive associations and contributes to theory-building by situating parental trust within the broader confidence component of behavioral models such as the 5C framework [10]. From this standpoint, greater trust corresponds with a higher likelihood that some parents reconsider previous refusals, indicating that trust could play an essential role in sustaining long-term vaccine acceptance.

From a practical perspective, this means that even if parents previously decided not to vaccinate a child, they should not be considered a “lost” group for preventive efforts. Consistent, calm, and empathetic communication was observed in other studies to be associated with greater parental trust and openness toward vaccination of subsequent children. At the systemic level, healthcare leaders must implement strategies supporting message consistency across the entire workforce, ensuring that parents do not receive contradictory signals from different professionals—a factor influencing vaccine hesitancy [8,45].

Finally, it is worth emphasizing that changing parental attitudes is a process that often requires time and repeated contact with the healthcare system. Therefore, during preventive or urgent visits, each interaction should be treated as an opportunity to strengthen relationships and encourage dialogue. In the long term, these efforts could align with lower vaccine hesitancy and greater vaccination coverage among children.

## 5. Limitations

This study includes several limitations that readers should consider when interpreting the findings. First, it employed a cross-sectional design, preventing a cause-and-effect relationship between variables. On the other hand, such a design can highlight important factors worth exploring in future research. Second, participant recruitment relied on purposive and snowball sampling, mainly through the internet and educational institutions, which likely overrepresented individuals who are more active on social media or more inclined to use digital communication channels. In addition, using a self-administered online questionnaire carries risks of declarative errors, response bias, and the inability to verify the information provided. Individuals with stronger views on vaccination were more inclined to participate, which may have influenced the results.

Another potential limitation concerns the Polish version of the PACV questionnaire. In the translation, the word “pediatrician” appeared in the last two questions, even though in the Polish healthcare system, routine child health monitoring and vaccination programs are primarily the responsibility of family physicians. This wording may have confused some participants, as noted in responses to the open-ended question at the end of the survey.

## 6. Implications for Future Practice and Research

The findings indicate that the level of trust in physicians significantly influences parental attitudes toward vaccination. Parents with lower levels of trust more frequently express concerns about vaccine safety, report fears of adverse post-vaccination reactions, and experience greater difficulties in openly communicating their doubts. Routine use of the PACV scale is a valuable tool for the early identification of Vaccine-Hesitant Parents (VHPs) before the first vaccination visit. Such an approach would enable physicians to tailor better conversations and arguments and optimize the organization of vaccination appointments, for example, by extending the consultation time for parents requiring additional explanations.

From a clinical practice perspective, these observations also confirm the importance of strengthening physician–parent relationships based on trust and transparent communication. Incorporating this aspect into family physician educational programs could improve the effectiveness of pro-vaccination efforts.

Future research should focus on evaluating the effectiveness of incorporating PACV as a screening tool in daily practice, assessing long-term changes in parental attitudes depending on the level of trust in physicians and the quality of communication during visits. Further analyses could also compare different professional groups (e.g., children’s doctors, nurses) to better understand which communicative interactions have the most significant impact on reducing vaccine hesitancy. Additionally, research into communication strategies for healthcare professionals could strengthen their ability to conduct conversations with vaccine-hesitant parents.

Additionally, future longitudinal studies could explore how trust and vaccination behaviors evolve between the first and subsequent children, providing deeper insight into the dynamics of parental decision-making.

## 7. Conclusions

Respondents believed that vaccinations protect against serious diseases. Although they expressed some reservations about the recommended vaccination schedule, they did not consider the number of vaccines administered to children excessively. The study confirmed a significant association between parental trust in physicians and the occurrence of hesitancy toward childhood vaccinations. This finding underscores the importance of the physician–patient relationship and communication quality as factors associated with parental vaccination attitudes. Significantly, parental attitudes toward vaccination may change with the arrival of additional children, indicating that earlier experiences with the healthcare system and vaccination are related to later parental decisions.

The PACV scale may serve as a valuable tool in clinical practice for the early identification of parents who require additional informational and communicative support, facilitating physician–parent dialogue and potentially aligning with higher vaccine acceptance among parents. These conclusions should be interpreted in light of the study’s cross-sectional and non-representative sample, which consisted mainly of digitally active, highly educated Polish mothers.

## Figures and Tables

**Table 1:** Characteristics of the Participants and Children.

Parents		Total (N = 1046)
Parent’s age [years]	Mean (SD)	36.56 (6.17)
	Median (quartiles)	36 (32–40)
	Range	20–60
	N	1041
Place of residence	Large city	454 (43.40%)
	Suburbs/outskirts of large city	147 (14.05%)
	Medium or small town	229 (21.89%)
	Village	194 (18.55%)
	Single household or house located in rural area	22 (2.10%)
Education	Primary	2 (0.19%)
	Vocational	15 (1.43%)
	Secondary	129 (12.33%)
	Higher	900 (86.04%)
Employment status	Unemployed	112 (10.71%)
	Employed	934 (89.29%)
Employment in healthcare	No	755 (72.18%)
	Yes	291 (27.82%)
Household size	1 person	1 (0.10%)
	2 persons	47 (4.49%)
	3 persons	425 (40.63%)
	4 persons	426 (40.73%)
	5 persons	118 (11.28%)
	6 or more persons	29 (2.77%)
Number of children under 18 in the household	1 child	514 (49.14%)
	2 children	428 (40.92%)
	3 children	87 (8.32%)
	4 children	15 (1.43%)
	5 children	2 (0.19%)
Rising children	Together with another parent/guardian	980 (93.69%)
	Alone	66 (6.31%)
Children		Total (N = 1701)
Child’s age [years]	Mean (SD)	6.48 (4.9)
	Median (quartiles)	5 (3–10)
	Range	0–33
	N	1693
Relationship	Mother	1552 (91.24%)
	Father	141 (8.29%)
	Stepmother	1 (0.06%)
	Stepfather	1 (0.06%)
	Sister	2 (0.12%)
	Legal guardian	4 (0.24%)

**Table 2:** Characteristics of the PACV Index.

General Characteristics of the PACV Index									
**PACV—Score**			Interpretation					N	%
0–50			Positive attitudes					1447	85.07%
51–100			Negative attitudes					254	14.93%
Descriptive Characteristics of the PACV Index									
Score range	N	Missing data	Mean	SD	Median	Min	Max	Q1	Q3
0–100	1701	0	25.10	24.12	16.67	0	100	6.67	36.67

**Table 3:** PACV score—univariate and multivariate logistic regression analyses.

Characteristic		N	Positive Responses	Univariate Models				Multivariate Models			
				OR	95%CI		*p*	OR	95%CI		*p*
Parent’s age [years]		-	-	0.984	0.962	1.008	0.187	0.995	0.963	1.028	0.76
Place of resident	Large city	710	595	1	ref.			1	ref.		
	Suburbs/outskirts of a large city	250	219	1.365	0.892	2.09	0.152	1.614	1.037	2.513	0.034 *
	Medium or small town	369	318	1.205	0.844	1.722	0.305	1.351	0.93	1.963	0.114
	Village	328	274	0.981	0.689	1.397	0.914	1.125	0.775	1.633	0.535
	Single household or house in rural area	44	41	2.641	0.804	8.675	0.109	4.188	1.237	14.181	0.021 *
Education	Primary	4	3	1	ref.						
	Vocational	27	14	0.359	0.033	3.901	0.4				
	Secondary	196	162	1.588	0.16	15.734	0.693				
	Higher	1474	1268	2.052	0.212	19.819	0.535				
Employment status	Unemployed	201	150	1	ref.			1	ref.		
	Employed	1500	1297	2.172	1.53	3.084	<0.001 *	1.869	1.284	2.721	0.001 *
Employment in healthcare	No	1230	1009	1	ref.			1	ref.		
	Yes	471	438	2.907	1.983	4.262	<0.001 *	2.785	1.872	4.144	<0.001 *
Household size (number of persons)		-	-	0.693	0.597	0.805	<0.001 *	0.791	0.577	1.085	0.146
Number of children under 18 in the household		-	-	0.677	0.579	0.792	<0.001 *	0.806	0.58	1.121	0.2
Child’s age [years]		-	-	0.979	0.953	1.005	0.114	0.974	0.94	1.009	0.148
Raising children	With parent/guardian	1622	1377	1	ref.						
	Alone	79	70	1.384	0.682	2.807	0.368				

* Statistically significant association (*p* < 0.05).

**Table 4:** Declarations about the child’s current, planned and delayed vaccinations.

	Have You Ever Delayed Having Your Child Get a Shot for Reasons Other Than Illness or Allergy? (Q13)		Have You Ever Decided Not to Have Your Child Get a Shot for Reasons Other Than Illness or Allergy? (Q14)		If You Had Another Infant Today, Would You Want Him/Her to Get All the Recommended Shots? (Q15)	
	N	%	N	%	N	%
Yes	119	11.4	114	10.9	921	88.0
No	881	84.2	921	88.0	61	5.8
Varies depending on the child	46	4.4	3	0.3	-	-
Not sure	-	-	8	0.8	64	6.1

**Table 5:** Trust in the child’s doctor and the recommended vaccination schedule—descriptive statistics.

Item	Mean	Median	SD	Min	Max
How sure are you that following the recommended shot schedule is a good idea for your child? (Q15) (0–10 scale) ^1^	8.28	10.00	2.721	0	10
All things considered, how much do you trust your child’s doctor? (Q27) (0–10 scale) ^2^	7.80	8.00	2.214	0	10

^1^ 0 (Not at all sure) to 10 (Completely sure); ^2^ 0 (Do not trust at all) to 10 (Completely trust).

**Table 6:** Relationship between past decisions on childhood vaccination and future intentions, with descriptive statistics (differences confirmed by U Mann–Whitney test) on trust in child’s doctor and the recommended vaccination schedule in Groups A (for individuals who have previously decided not to vaccinate but plan to vaccinate in the future) and B (for individuals who have previously decided not to vaccinate and still do not plan to vaccinate).

			Have You Ever Decided Not to Have Your Child Get a Shot for Reasons Other Than Illness or Allergy? (Q14)							
			Yes				No			
If you had another infant today, would you want him/her to get all the recommended shots? (Q23)			N		%		N		%	
	Yes		45 (Group A)		39.5		870		94.5	
	No		47 (Group B)		41.2		113		1.4	
	Not sure		22		19.3		38		4.1	
	Total		114		100		921		100	
	Group A (N = 45)					Group B (N = 47)				
	mean	median	SD	min	max	mean	median	SD	Min	max
All things considered, how much do you trust your child’s doctor? (Q27)	7.49 ^1^	8.00	2.283	0	10	3.74 ^1^	4	2.786	0	10
How sure are you that following the recommended shot schedule is a good idea for your child? (Q15)	7.89 ^2^	9.00	2.376	0	10	1.40 ^2^	0	2.223	0	9

^1^ The mean difference was statistically significant (Mann–Whitney U = 304.0; *p* < 0.001). ^2^ The mean difference was statistically significant (Mann–Whitney U = 101.5; *p* < 0.001).

## Data Availability

The datasets used and/or analyzed during the current study are available from the corresponding author upon reasonable request.

## Abbreviations

The following abbreviations are used in this manuscript:

WHO	World Health Organization
COVID-19	Coronavirus Disease 2019
PACV	Parent Attitudes about Childhood Vaccines
MMR	Measles, Mumps, and Rubella
AEFI	Adverse Event Following Immunisation

## Appendix A

**Table A1:** Study questionnaire based on PACV.

Item		Response Format
Q1.	How old are you?	Numeric entry
Q2.	What is your nationality?	Polish/Ukrainian/Other
Q3.	Which of the following best describes where you live?	Large city/Suburbs or outskirts of a large city/Medium or small town/Village/Single household or house in a rural area
Q4.	What is your level of education?	Primary/Vocational/Secondary/Higher
Q5.	What is your employment status	Employed/Unemployed
Q6.	Do you work in healthcare?	Yes/No
Q7.	Number of people in your household	Numeric entry
Q8.	Number of children under 18 in your household	Numeric entry
Q9.	Age of child	Numeric entry
Q10.	I raise my child/children	Together with another parent or guardian/Alone
Q11.	Net monthly household income	Numeric entry
Q12.	What is your relationship to the child/children?	Mother/Father/Legal guardian
Q13.	Have you ever delayed having your child get a shot for reasons other than illness or allergy?	Yes/No/Don’t Know
Q14.	Have you ever decided not to have your child get a shot for reasons other than illness or allergy?	Yes/No/Don’t Know
Q15.	How sure are you that following the recommended shot schedule is a good idea for your child?	0 (Not at all sure) to 10 (Completely sure)
Q16.	Children get more shots than are good for them.	** SA/A/NS/D/SD
Q17.	I believe that many of the illnesses shots prevent are severe.	** SA/A/NS/D/SD
Q18.	It is better for my child to develop immunity by getting sick than to get a shot.	** SA/A/NS/D/SD
Q19.	It is better for children to get fewer vaccines at the same time.	** SA/A/NS/D/SD
Q20.	How concerned are you that your child might have a serious side effect from a shot?	*** NAC/NTC/NS/SC/VC
Q21.	How concerned are you that any one of the childhood shots might not be safe?	*** NAC/NTC/NS/SC/VC
Q22.	How concerned are you that a shot might not prevent the disease?	*** NAC/NTC/NS/SC/VC
Q23.	If you had another infant today, would you want him/her to get all the recommended shots?	Yes/No/Don’t Know
Q24.	Overall, how hesitant about childhood shots would you consider yourself to be?	* NAH/NTH/NS/SH/VH
Q25.	I trust the information I receive about shots.	** SA/A/NS/D/SD
Q26.	I am able to openly discuss my concerns about shots with my child’s doctor.	** SA/A/NS/D/SD
Q27.	All things considered, how much do you trust your child’s doctor?	0 (Do not trust at all) to 10 (Completely trust)
Q28.	Comments field	Open text

* NAH/NTH/NS/SH/VH—Not at all hesitant/Not too hesitant/Not sure/Somewhat hesitant/Very hesitant. ** SA/A/NS/D/SD—Strongly Agree/Agree/Not sure/Disagree/Strongly Disagree. *** NAC/NTC/NS/SC/VC—Not at all concerned/Not too concerned/Not sure/Somewhat concerned/Very concerned.
